# Attachment Theory in Adult Mental Health: A Guide to Clinical Practice

**DOI:** 10.1192/pb.bp.115.052662

**Published:** 2016-12

**Authors:** Jon Patrick

In the opening chapter of this tour through attachment theory, Jeremy Holmes suggests a good question to ask patients: ‘Who would you contact first if there was an emergency or crisis in your life?’ This is a great way to get to the bottom of who your attachment figure is. Interestingly, during my read of this book I found myself thinking – ‘which chapter will I turn to for support with my clinical problems?’

Some chapters were more clear and mindful of their audience than others. I particularly enjoyed the introduction to the concepts of attachment theory as well as the chapters on psychosis, personality disorder and eating disorders. The latter – with their use of vignettes – were especially effective at helping me conjure up actual scenarios and reminded me of how valuable attachment theory is as a way of conceptualising therapeutic encounters. On the other hand, some chapters had problems maintaining narrative coherence. The difference was often determined by whether the authors used clinical material to enliven theory. Perhaps because multiple authors were involved, attachment theory was explained repeatedly at the start of most chapters, which left me irritated and less responsive. Although the editors suggest that readers might dip into the book, I found these theoretical recaps tiring.

What the book does really well is demonstrate how Bowlby's theory has allowed disparate paradigms to coexist. The influence and provision of a common language, for thinkers from scientific backgrounds as distant as evolution and psychoanalysis, is impressive to see here. There may be technical disagreements but they are usefully explored by contrasting the different approaches outlined in the book, from neurocognitive to cognitive-behavioural to mentalisation-based.

This guide, with its many helpful ideas, illustrates the need to consider patients from cradle to grave and how attachment thinking is relevant across contexts, be these diagnostic, cultural or systemic. The penultimate chapter by Seager, about using attachment theory to inform services, is particularly relevant given current reorganisation of some areas within the National Health Service, where sometimes it seems people and their relationships are being forgotten. He asks a powerful question which has made me reflect seriously about my work: ‘If early attachment relationships are this powerful and formative in human personality development, why is our society so blind to attachments when designing its mental health care systems?’

